# Editorial: Minding the Gradient — Valve-in-Valve Transcatheter Aortic Valve Replacement vs. Redo Surgical Aortic Valve Replacements for Surgical Bioprosthetic Valve Failure

**DOI:** 10.1016/j.shj.2022.100113

**Published:** 2022-10-27

**Authors:** Syed Zaid, Neal S. Kleiman

**Affiliations:** Department of Cardiology, Section of Interventional Cardiology, Houston Methodist DeBakey Heart and Vascular Center, Houston, Texas, USA

More than 60% of the 60,000 surgical aortic valve replacements (SAVRs) performed annually in the United States involve bioprosthetic valves.^1^ While the 10-year rate of clinically relevant structural valve deterioration after SAVRs has been estimated to be between 5% and 15%,^2^ its true frequency is likely underestimated. Redo-SAVR has been the conventional treatment for surgical bioprosthetic valve failure. However, the complexity of a redo-surgery is associated with higher early morbidity and mortality than a first-time SAVR.^3^ Accordingly, valve-in-valve (ViV) transcatheter aortic valve replacement (TAVR) has emerged as a promising alternative and was approved by the Food and Drug Administration in 2015 for patients at prohibitive or high surgical risk of a redo-SAVR. A systematic review of early studies revealed short-term benefits of ViV TAVR over redo-SAVR.^4^ Several preliminary studies have subsequently revealed a good intermediate-term valve durability.^5^, ^6^, ^7^

While ViV TAVR is an attractive option, how a transcatheter heart valve (THV) within a retained surgical bioprosthesis will behave within a broad population, particularly with regard to hemodynamic performance and long-term clinical outcomes, is not yet known. The ViV TAVR procedure poses 2 important anatomic/technical challenges. First, coronary obstruction has been reported in about 2% of ViV procedures^5^; however, careful patient screening and electrocautery leaflet laceration may have minimized this problem. The second and more common challenge is that of elevated postprocedural gradients. THV underexpansion is common in ViV implants, leading to higher residual gradients, and is influenced by the mode of SAV failure, bioprosthetic SAV size and type of SAV, and the type of TAV selected. Four studies have thus far reported intermediate-term findings among patients at an intermediate or a higher risk of undergoing ViV TAVR with mortality estimates ranging from 28.6% at 3 years to 62% at 8 years.^5^, ^6^, ^7^, ^8^

In the current issue of the *Structural Heart*, 2 additional manuscripts report outcomes of ViV TAVRs. First, a single-center observational analysis by Hecht et al. compares hemodynamic and long-term clinical outcomes between patients undergoing ViV TAVR and those undergoing a redo-SAVR.^9^ The investigators studied 184 patients treated for surgical bioprosthetic valve failure, with redo-SAVR or ViV TAVR, between 2009 and 2017 (62.5% with Sapien (Edwards Lifesciences, Irvine, CA) valves and the remainder with CoreValve (Medtronic, Minneapolis, MN) or Portico (Plymouth, MN) valves). The median follow-up duration was 5.6 years. Compared with redo-SAVR, ViV TAVR resulted, respectively, in a higher residual mean gradient (22.1 ± 9.3 mm vs. 18.2 ± 9.4 mm), more frequent severe prosthesis-patient mismatch (PPM) (59.5% vs. 41.7%), and a lower rate of intended hemodynamic performance (39.2% vs. 67.7%), driven primarily by a more frequent residual mean gradient >20 mmHg. At 30 days, ViV TAVR trended toward lower mortality (2.5% vs. 8.7%, *p* = 0.08), but unlike the comparison performed in the REDO-TAVR Registry,^8^ ViV TAVR was independently associated with an increased risk of long-term mortality (hazard ratio 2.06 [1.06-3.88], *p* = 0.025). Damning as these observations may seem for ViV TAVR, several critical points must be kept in mind. First, in the current report, mortality was nearly 40% for ViV TAVR, compared with the approximately 20% rate reported in the REDO-TAVR registry,^8^ suggesting that patients in the present study may have had a greater acuity. In fact, compared to redo-SAVR, ViV TAVR patients were older, more likely to have small (<21 mm) surgical prostheses (32.5% vs. 12.5%), and to have pre-existing PPM (39.1% vs. 22.5%). Furthermore, 21.1% of surgical patients underwent enlargement or replacement of the aortic root, and a large proportion underwent other surgical valve procedures, strongly suggesting that they were judged to be at a lower surgical risk. Although the authors attempted to adjust for these differences using inverse probability weighting, it seems unlikely that even with this technique, fully comparable groups could be constructed.

In the second paper, Malaisrie et al. report more contemporary outcomes after ViV TAVR in low- to intermediate-risk patients.^10^ In a prospective, single-arm, multicenter study, the investigators studied 97 patients (out of 165 patients screened) who underwent ViV TAVR using Sapien 3 valves between 2017 and 2019. In this younger, lower-risk cohort (mean age 67 years, Society of Thoracic Surgeons (STS) Predicted Risk of Mortality 2.9%), there were no deaths at 1 year, and stroke occurred in 2 patients. However, 5 patients experienced valve thrombosis, and 9 were rehospitalized, 6 of whom had an aortic valve reintervention. At 1 year, there were sustained improvements in New York Heart Association functional class and hemodynamic status (mean gradient 19.7 ± 7.6 mmHg; >moderate aortic regurgitation in 1.1%). Complete follow-up is planned for 10 years.

The most noteworthy feature of both studies is that residual gradients at 30 days were high following ViV TAVR. The 22.1-mm gradient reported by Hecht et al. and the 22-mm gradient reported by Malaisrie et al. are considerably higher than those typically observed after TAVRs in native valves and are even higher than the 15 to 18 mmHg previously reported for ViV TAVRs.^6^, ^7^, ^8^ Such figures often cause considerable consternation to TAVR operators as the entire purpose in replacing a narrowed aortic valve is to eliminate the transvalvular gradient. Although large-scale studies relating the mean gradient to clinical outcomes are difficult to find, there is a substantial body of literature concerning PPM, which is a measure of transvalvular gradient indexed for patient size. In the STS registry, the frequency of severe PPM (calculated based on the reference diameter for each valve) among 59,779 patients undergoing SAVRs was 11%. After 10 years, any degree of PPM decreased long-term survival; severe PPM was associated with a 32% increase in adjusted mortality.^11^ In a report from the STS/American College of Cardiology transcatheter valve therapy registry, severe PPM was present in 12% of 62,125 patients who underwent TAVR and was associated with a 19% increase in mortality at 1 year.^12^ It is notable that the analyses in the 2 current reports seem to belie these findings. Hecht et al. found no association between a high residual gradient, severe PPM, or failed intended hemodynamic performance and long-term mortality, while Malaisrie et al. reported that left ventricular mass regression at 1 ​year was nearly identical between patients with gradients <20 mmHg and those with gradients >20 mmHg. These surprising and counterintuitive observations are actually in line with previous reports of THV in SAV. In the “CoreValve Extreme Risk ViV” study, hemodynamic parameters including the mean gradient were not independent predictors of 3-year mortality,^6^ and the PARTNER Sapien XT investigators also found that neither PPM nor initial surgical valve size (a surrogate for post-TAVR gradient) independently predicted mortality at 5 ​years although the use of the 23-mm rather than the 26-mm Sapien XT was a predictor.^7^ Similar observations were made in the Valve-in-Valve International Data registry.^13^

How are we to interpret these data? Intuitively, one would expect that higher gradients would be associated with reduced survival and that operators should aim for the lowest possible gradient. If the relationships observed for TAVR or SAVR in native valves are not truly applicable to ViV TAVR, then these observations might call into question such practices as valve fracture^14^ or selecting supra-annular valves for ViV TAVR because of their inherently lower gradients.^15^ With about 60,000 SAVRs/y performed in the United States over the last decade, one can calculate that >350,000 patients have bioprosthetic aortic valves. On the other hand, the observations that clinical and hemodynamic outcomes are better when a ViV TAVR is performed in larger rather than smaller valves may be a signal that a similar relationship holds for ViV TAVRs as in the native-valve TAVR. The statistical certainty of these observations is limited. The 4 largest ViV TAVR studies with long- or intermediate-term follow-up contain a total of 1212 patients compared with >120,000 in the STS and transcatheter valve therapy registries. It is easy to argue then that they are underpowered. Perhaps the range of post-ViV TAVR gradients is too limited to make meaningful distinctions between high and low gradients. It seems too early to abandon the aim of reducing the post-ViV TAVR gradient as aggressively as is safe. As we await the accumulation of more robust data, we should at least remain circumspect about selecting ViV TAVR in patients with small surgical valves (internal diameter <21 mm internal diameter) or preexisting PPM, particularly at a time when aortic root enlargement at the time of redo-SAVR is being explored.

With an average estimated longevity of 10-12 years per bioprosthesis, many patients should soon be approaching the need for further valve intervention. (Parallel questions will soon arise for patients who have undergone native-valve TAVR.) These challenges will require dedicated solutions from both operators and device manufacturers alike. It is an established trope among academic physicians to call for randomized trials. There is no doubt that performing such a trial would be arduous and considerably more difficult than studying TAVR in native valves. However, the growing wave of patients with SAVR approaching the end of valve life and the directionally opposite results reported by Hecht et al. and the REDO-TAVR registry suggest that there is considerable equipoise. The development of valve-modification procedures and surgical aortic root enlargement procedures and surgical valves designed to facilitate later TAVR enhances that equipoise, while the report by Hecht et al. would suggest that the current procedures are at a sufficient level of maturity to support designing appropriate randomized trials with long-term follow-up.

## Funding

There was no funding for this editorial.

## Disclosure statement

Syed Zaid had nothing to disclose. Neal Kleiman is an investigator for Medtronic, Abbott, Boston Scientific, and Edwards.
